# Microstructure Evolution and Orientation Relationship of Reverted Austenite in 13Cr Supermartensitic Stainless Steel During the Tempering Process

**DOI:** 10.3390/ma12040589

**Published:** 2019-02-15

**Authors:** Yiwei Zhang, Chi Zhang, Xiaomin Yuan, Diankai Li, Yuande Yin, Shengzhi Li

**Affiliations:** 1 School of Materials Science and Engineering, Anhui University of Technology, Ma’anshan 243002, China; chizhang16@126.com (C.Z.); yuan@ahut.edu.cn (X.Y.); diankaili@126.com (D.L.); 2 School of Metallurgical Engineering, Anhui University of Technology, Ma’anshan 243002, China; yinyd@ahut.edu.cn

**Keywords:** supermartensitic stainless steel, reverted austenite, orientation relationship, electron backscattered diffraction

## Abstract

The transformation mechanism of reverted austenite and the amount of reverted austenite during the tempering process in supermartensitic stainless steel have been investigated by X-ray diffraction (XRD), electron backscattered diffraction (EBSD), and a high-temperature laser scanning confocal microscope (HTLSCM). The results indicate that the microstructure mainly consists of tempered martensite and reverted austenite. The reverted austenite nucleates uniformly at the sub-block boundary and prior grain austenite boundary. The amount of reverted austenite strongly relies on the tempering time, showing a positive correlation in the supermartensitic stainless steel. The crystallographic orientation relationship between reverted austenite and martensite meets the Kurdjumov-Sachs(K-S) relationship and the deviation angle is mainly concentrated at about 2 degrees. The mechanism of reverted austenite transformed from martensite is a diffusion mechanism. The growth kinetics of the reverted austenite are dominated by diffusion of the Ni element and there is no shear deformation of the martensite matrix in the in situ observation. It can be deduced that the reverted austenite is formed by nickel diffusion during tempering at 620 °C for different tempering times.

## 1. Introduction

Supermartensitic stainless steel has outstanding mechanical properties, including a high strength, good toughness, excellent corrosion resistance, and weldability [1,2,3,4,5]. It has such excellent mechanical properties that it is increasingly applied to the offshore oil and gas industry and other scopes of the Oil Country Tubular Goods (OCTG) market [6]. It is used in aggressive corrosion environments and offers improved corrosion resistance. Generally, it has such a low production cost that it can replace duplex stainless grades [7,8].

In industrial production processes, the hardness and strength values of supermartensitic stainless steel are of the utmost importance. The mechanical properties are usually controlled by chemical composition and thermo-mechanical treatments [9,10,11,12,13,14,15]. Nevertheless, they are often observed with various factors of the final mechanical properties. The mechanical properties are strongly dependent on the fraction of reverted austenite [16,17,18,19,20], which is very sensitive to the heat treatment. Minor changes in heat treatment parameters will result in significant changes in the mechanical properties. The heat treatments of supermartensitic stainless steel are achieved by quenching and tempering after hot working [21].

The microstructure is tempered martensite and reverted austenite produced by the quenching and tempering of supermartensitic stainless steel. The nanometer scale of reverted austenite is fine and dispersed in the tempered martensite matrix. The tempered martensite structure has been shown to supply a superior strength and excellent low temperature toughness owing to the reverted austenite [22,23]. The existence of the reverted austenite improves the excellent mechanical properties, especially ductility for the steel, and the amount and morphology of reverted austenite was controlled by tempering temperature and time [17,22].

Tempering processes are mandatory for supermartensitic stainless steel in order to achieve the desired microstructure. However, the amount, morphology, and distribution of reverted austenite are very sensitive to the tempering temperature and tempering time. Therefore, it is essential to study the microstructural characteristics, volume fraction, and formation mechanism of the reverted austenite in SMSS [24,25]. There are several factors that affect the stabilization of the reverted austenite in steel. The nickel and carbon elements are partitioned into the austenite and stabilize it to ambient temperature [26]. The substructure and prior austenite morphology have an effect on the stability of the reverted austenite [27]. Obviously, the stability of the reversed austenite is determined by no less than one factor.

The formation mechanism that transforms the martensite to the reverted austenite has been discussed for supermartensitic stainless steel in the literature [20,28,29,30,31,32], which has presented the diffusion mechanism and diffusionless shear mechanism. Previous research has proven that Ni is enriched in the reverted austenite and affects its stability [19,20,22,33], and the growth of the reverted austenite is the diffusion process of the Ni element. The tempering temperature was the main factor affecting the reverted austenite formation of SMSS. The transformation mechanism is still ambiguous and the research methods used are practically insufficient. This is because that crystallographic information is relatively microscopic and is only on a scale of one single austenite grain obtained by TEM.

In the present work, more precise study on the reversed austenite formation during tempering has been done using the electron backscattering diffraction (EBSD) technique. The effects of different tempering times on the reversed austenite formation are still unclear. It has been used to investigate the phase transformation distribution and examine the crystallographic orientation between reverted austenite and martensite with a large area covering prior austenite grain in SMSS. Additionally, the work was pursued to find the transformation mechanism of reverted austenite using a high-temperature laser scanning confocal microscope (HTLSCM) and the factors that influence the amount of the reverted austenite and mechanical properties.

## 2. Materials and Methods

Supermartensitic stainless steel was obtained by a commercial Super 13Cr and its composition is given in Table 1. The materials were hot rolled, followed by heat treatment and then air cooling to room temperature. The samples from a commercial 13Cr supermartensitic stainless steel were subjected to quenching at 1050 °C for 30 min in a resistance furnace, followed by tempering at 620 °C for 1 h, 2 h, 4 h, 8 h, 16 h, and 32 h by air cooling to ambient temperature. To better characterize the formation of reversed austenite during the α→γ transformation, austenite nucleation and growth characteristics from martensite were observed in situ by a high-temperature laser scanning confocal microscope (HTLSCM) (YONEKURA MFG CO., LTD, Osaka, Japan). The microstructure during the heating and holding process was observed and stored as a video file by a high-temperature laser scanning confocal microscope (HTLSCM), which is a rather new technique for the in situ analysis of high temperature phase transformation and microstructure evolution [31,34]. The specimens were machined to round bars of 8 mm diameter × 3 mm thickness and the samples were oil quenched at 1050 °C for 30 min. One side of the disc specimen was mechanically polished for observation before being mounted in a high purity alumina crucible. The specimen was heated to 620 °C at the rate of 5 °C/s and held at this temperature for 60 min. Heating, holding, and observation of the α→γ transformation under HTLSCM were carried out in a vacuum of about 1.33 × 10^−2^ Pa. Microstructure development during the heating and tempering holding process was then observed and stored as a video file.

The specimens was etched by Vilella’s etchant, which contained 5 mL hydrochloric + 4 g picric acid +100 mL ethyl alcohol. Microstructural characterization was determined by optical microscopy (CarlZeiss, Jena, Germany) and JEOL6490 scanning electron microscopy (JEOL, Tokyo, Japan). The crystallographic orientation relationship between reverted austenite and martensite matrix was characterized by electron backscattered diffraction (EBSD) (Oxford, London, UK). The EBSD specimens were polished with a 3 μm diamond suspension for 60 min and a 1μm diamond suspension for 60 min. Finally, the colloidal silica solution slightly etched the sample during the mechanical polishing process to remove relief. Ideally, the samples should be polished for 10 min to several hours with colloidal silica. The EBSD maps of tempering samples were obtained using a high resolution microscope FEI-Nano 430 (FEI, Hillsboro, OR, USA) operated at 20 kV and with a 200 nm step size.

The reverted austenite in the tempered samples was measured at room temperature by X-ray diffraction using Cu-Kα radiation from 30 to 100° at a 0.02° step interval and the exposition time of 0.3 s per step. The volume fraction of reverted austenite was quantitatively measured by the Bruke D8 X-ray diffraction instrument using a Cu X-ray source operated at 40 kV and 40 mA. The calculations were based on the integrated intensities of (110)α, (200)α, and (211)α martensite peaks, as well as the (111)γ, (200)γ, and (220)γ austenite diffraction peaks, through the following equations [17,35,36]:

(1) $$V_{\gamma }+V_{\alpha }=1$$

(2) $$V_{\gamma }=1.4I_{\gamma }/\left(I_{\alpha }+1.4I_{\gamma }\right)$$

where *V*_α_ and *V*_γ_ are the volume fraction of martensite and reverted austenite, respectively. *I*_α_ and *I*_γ_ are the average integral intensities of the martensite and austenite peaks, respectively.

The mechanical properties of the heat treatment specimens were subjected by means of micro-hardness. The hardness tests were evaluated by Rockwell Hardometer Vickers (Weiyi Testing Instrument Co., Ltd., Laizhou, China) using a load of 98 N and a 15 s dwell time. Each value was the average of hardness values at ten points.

## 3. Results and Discussion

### 3.1. Microstructure Characterization of Reverted Austenite

**Figure 1:**
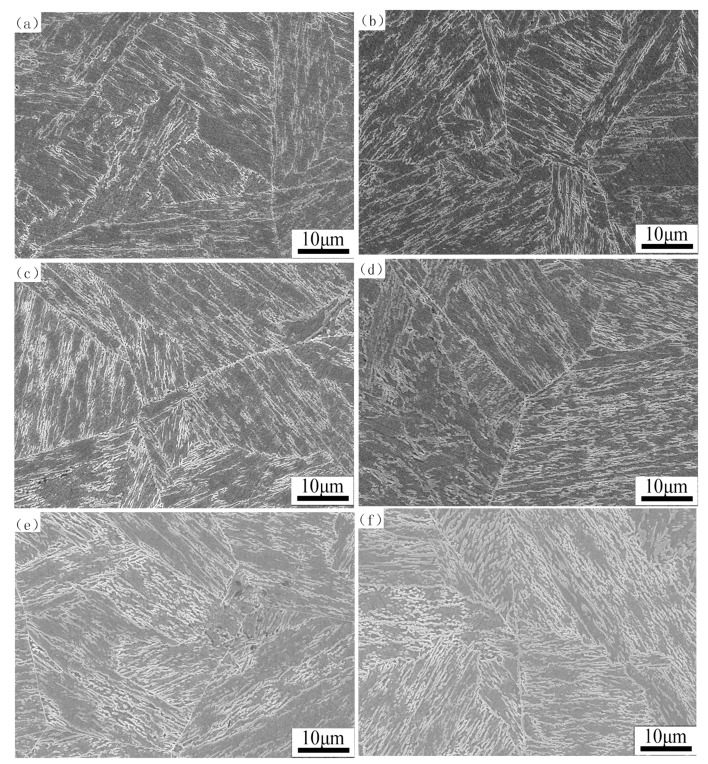
SEM microstructure of the samples tempered at (**a**) 1 h, (**b**) 2 h, (**c**) 4 h, (**d**) 8 h, (**e**) 16 h, and (**f**) 32 h.

The multiphase microstructure of samples tempered at 620 °C for different times is shown in Figure 1. There is low carbon tempered martensite and reverted austenite in the microstructure of supermartensitic stainless steel. It can be seen that the martensite exhibiting lath morphology and reverted austenite formed at the laths in the micrographs. It seems that the reversed austenite was transformed at α phase lath boundaries and inside the laths. When the tempering time is increased from 1 h to 32 h, the martensite lath width gradually becomes narrowed and the reverted austenite content increases remarkably. As the tempering time increases, the inter-granular dislocation density of lath tempered martensite decreases and the martensite is decomposed; meanwhile, the internal stress of the microstructure is gradually released, since the reverted austenite is arranged like platelets among martensite laths [19,27]. In previous research [37], the carbon element was preferentially positioned in the reverted austenite, it was not precipitated in alloy carbides in the matrix during tempering, and the martensite was kept in the shape of laths. Subsequently, a coalescence of the reverted austenite appeared.

### 3.2. The Effect of Reverted Autenite Contents on Mechanical Properties

**Figure 2:**
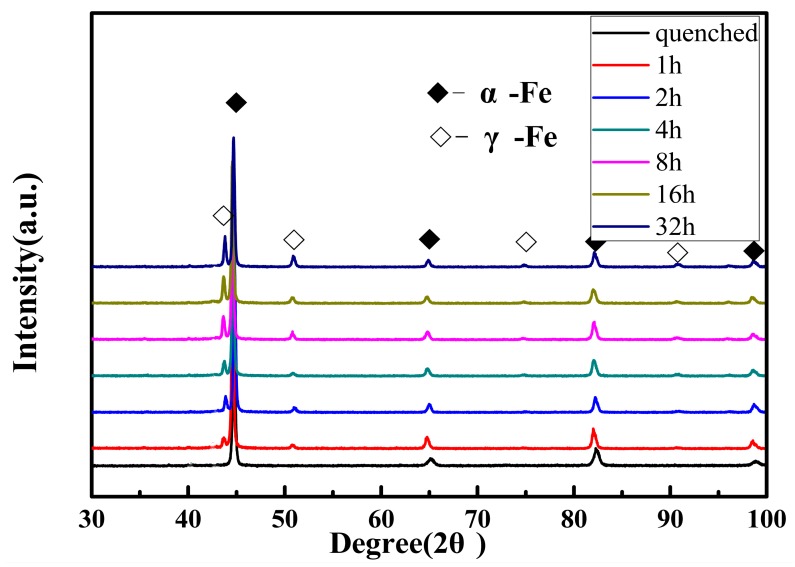
XRD pattern of the samples quenched and tempered at 620 °C for different tempering times.

The reverted austenite content determines the properties of the supermartensitic stainless steel [38]. Therefore, it is critical to precisely measure the reverted austenite contents after different tempering processes of supermartensitic stainless steel. It is guaranteed that the reverted austenite is transformed from martensite in the tempering process, rather than being retained as austenite in the quenched sample. The XRD pattern of supermartensitic stainless steel after quenching and then tempering at 620 °C for different tempered times is shown in Figure 2. The XRD pattern figure indicated that there are only the α-Fe and γ-Fe phases in the samples of different tempering times. However, there are no γ-Fe phase peaks in the quenched sample, which reveals that the content of the retained austenite is close to zero. This means that there is no retained austenite after quenching at 1050 °C. In Figure 2, the black squares represent martensite and the white squares represent reverted austenite, and no texture exists in the samples. However, the intensity of the (111) crystal plane varies greatly. This represents that reverted austenite contents are different in the supermartensitic stainless steel for different tempering times.

**Figure 3:**
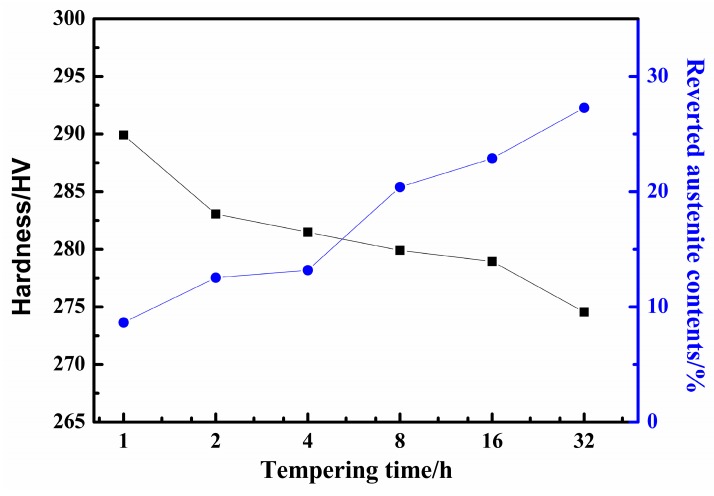
The variation of micro hardness and reverted austenite tempered at 620 °C for different tempering times.

The XRD pattern indicated that the volume fractions of reverted austenite are different in samples as the tempering time increased from 1 h to 32 h. According to the Equations (1) and (2), the results revealed that the abundance of reverted austenite phase was 8.7%, 12.5%, 13.2%, 20.4%, 22.9%, and 27.3%, respectively. The amounts of reverted austenite show a significant upward trend according to the results of the XRD pattern of the supermartensitic stainless steel for different tempering times. It was found that the reverted austenite content increased gradually when the tempering time increased from 1 h to 32 h, as shown in Figure 3. The results on reverted austenite contents are in good agreement with other experiments [17,22]. The volume of reverted austenite could be attributed to the austenite stabilization elements [39]. The austenization stability elements of partitioning C and Ni are enhanced as the tempering time increases. The result of the concentration of C and Ni elements is that the Ms point is lowered and the stability of the reverted austenite is increased, leading to an increasing amount of reverted austenite [9,13].

Figure 3 represents the variation in micro-hardness and the contents of reverted austenite tempered at 620 °C for different tempering times. It manifested that the average hardness gradually decreases from 290 HV to 275 HV as the tempering time increases. There is a remarkable decrease in the hardness with the increasing tempering time, which is marked on the black dot line. However, the amounts of reverted austenite marked on the blue dot line had an opposite tendency with the tempering time. It was anticipated that the value must be the maximum amount of austenite and the hardness reaches the minimum value when the specimens were tempered at 620 °C for 32 h in the steel. It is quite obvious that the amount of reversed austenite gradually increases with tempering time. The trend in the hardness decreased, which can be attributed to the amount of reverted austenite. Additionally, the average grain size became coarser with an increasing tempering time. The amounts of reverted austenite are dependent on grain size, along with chemical elements enrichment. The C and Ni elements are steadily partitioned into austenite with increasing tempering time, thus resulting in a higher volume fraction of reverted austenite. is the results also indicated that the hardness decreases with an increasing tempering time [39].

The reverted austenite as a soft phase after tempering does not retain the high density dislocation structure [18]. As the tempering time increases, the concentration of the quenching vacancies becomes lower in the microstructure, reducing the strength and hardness. The result reveals the strong dependence of mechanical properties on the tempering processes.

### 3.3. The Misorientation Analysis of the Reverted Austenite

**Figure 4:**
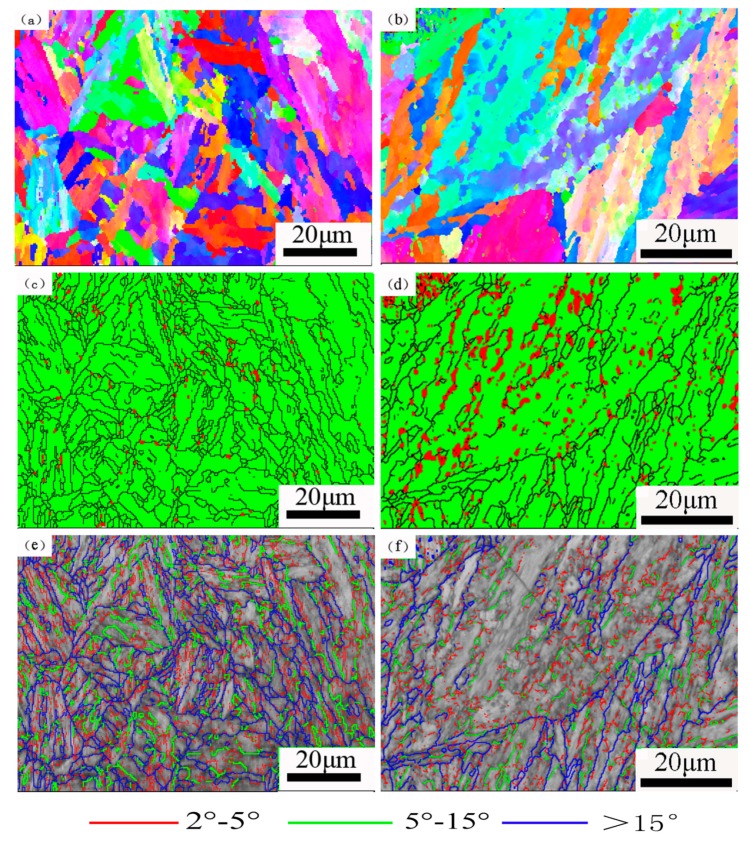
EBSD analysis results of the samples tempered for 2 h and 32 h, respectively: (**a**,**b**) the inverse pole figure map of reverted austenite and martensite; (**c**,**d**) phase distribution of martensite and reverted austenite; (**e**,**f**) high and low angle grain boundaries with misorientation angles.

The microstructure of the samples tempered at 620 °C for 2 h and 32 h and characterized by EBSD is shown in Figure 4. The color represents the crystallographic orientation in Figure 4a,b. It can be seen that there are multiple martensite crystallographic orientations within the prior austenite grains. The martensite structure consists of packets and blocks of different colors and orientations.

The micrographs of phase distribution show that the reverted austenite phase is red colored and the martensite phase is green colored. It is obvious that the plated reverted austenite primarily located in the region of lath martensite, and some are distributed in the prior austenite grain boundaries. It can be seen that the reverted austenite content of tempering time for 32 h is much greater than tempering for 2 h. It can be regarded that the reverted austenite phases, as a plate-like shape or a globular morphology, distribute in the martensitic lath boundaries and prior austenite grain boundaries, and are sometimes concentrated in the phase interface [24,27,30,40].

The orientation angles distribution in the martensite structure is shown in Figure 4e,f. The large angle boundaries with misorientation larger than 15° are depicted as thick blue lines. Generally speaking, the prior austenite grain boundary orientation angle is greater than 15° [30]. The low angle sub block boundaries in martensite were marked by green lines with misorientations in the range of 5° to 15°, whereas red lines mark small angle grain boundaries with misorientations that range from 2 to 5°. All the laths of martensite have crystallographic and morphological features with the misorientation range of 0~60° in low carbon steel and the angle of the martensite sub-block is about 10° [41]. It was found that the low angle martensite sub block boundaries are significantly reduced and the small angle grain boundaries are also decreased in the tempered microstructure. Meanwhile, the internal stress of the microstructure is released and the defect density is decreased. Finally, the prior austenite grains grow coarse after a long period of tempering and the microstructure tends to be homogenized.

**Figure 5:**
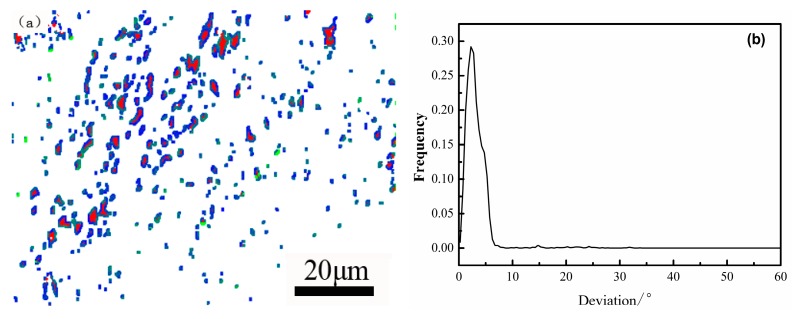
(**a**) K-S orientation relationship of bcc and fcc phase of the supermartensitic stainless steel and (**b**) the delivation angle of the ideal K-S orientation relationship boundaries.

Figure 5 is the K-S orientation relationship of the bcc and fcc interface map and the deviation angle of the ideal K-S orientation relationship of the supermartensitic stainless steel. An identical orientation K–S relationship is seen for the martensite matrix and reverted austenite: plane (111)γ//(011)α and direction [1$\bar{1}$0]γ//[1$\bar{1}$1]α [24,42]. In Figure 5a, the blue color represents the ideal K-S relationship angle that is more than zero degrees, and the green color is the deviation angle greater than 10 degrees. It is shown that the martensite phases colored white have good interface distribution characteristics with red reverted austenite phases. In Figure 5b, it can be conspicuously seen that martensite and reverted austenite satisfy the K-S orientation, and the deviation angle is mainly concentrated at about 2 degrees. According to Grajcar et al. [43], it is estimated that a fraction of grain boundaries fulfilling the K-S relationship within 10 degrees of the deviation angle is approximately 98.4%. It is verified that the result on the K-S orientation relationship obtained by EBSD has universal applicability by TEM technology. The analysis results are also consistent with the theoretical calculations in the literature [44].

**Figure 6:**
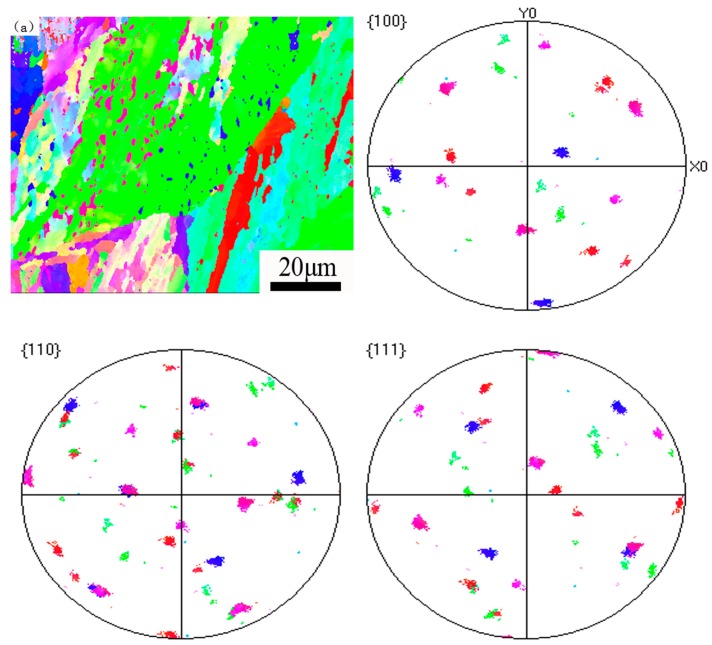
Crystallographic orientation maps and inverse pole figure of the martensite matrix and reversed austenite and (100), (110), (111) pole figure of the reverted austenite showing orientations corresponding to the EBSD orientation map.

The orientation distribution of reverted austenite was investigated by a pole map in order to discuss orientation analysis. In Figure 6, there are (100), (110), and (111) pole figures showing orientations of the reverted austenite corresponding to the EBSD orientation map, respectively. The reverted austenites with different colors have the same orientation as the surrounding martensite, and satisfy the K-S orientation distribution characteristic. The different colors of reverted austenites indicate that there are different close packed planes and close packed directions [24]. It considers that the nucleation position and growing mechanism of the other color reversed austenite are different from the reverted austenite [11,25]. Therefore, we showed that the growing mechanism of this kind of reverted austenite is a diffused phase transformation, and the growth of the reverted austenites is closely related to the diffusion process of Ni.

These maps reveal that reverted austenite grains are uniformly formed not only on lath boundaries, but also on packet boundaries and block boundaries.

### 3.4. The mechanism of the Reverted Austenite

There are diffusionless shear mechanisms and diffusion mechanisms of the reverted austenite. The mechanism of reverted austenite formation has been explored by many works on supermartensitic stainless steel [28]. It is accepted that many factors, such as composition, heating rate, holding temperature, and holding time, determine the reverted austenite formation behavior [18,23,29,37]. It can be observed that the higher the tempering time, the greater the amount of reversed austenite [22].

**Figure 7:**
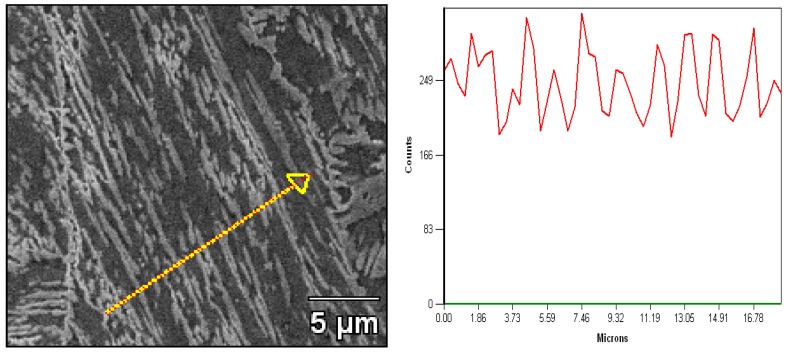
SEM micrograph of the samples tempered at 620 °C for 32 h and the Ni element distribution of line scan spectrum EDX in the microstructure.

It was shown that the Ni element distributed in the microstructure of the line scan energy diffraction spectrum in Figure 7. The tempered martensite matrix is a light gray area and the reverted austenite is represented by a white color with a strip or plate shape. The line scan results of EDX show that the Ni element contents appear as unevenness trends in the curve, indicating that the Ni distribution is different in the two structures. At the same time, the nickel content of the reverted austenite appears to be a peak value, but the matrix around the reverted austenite appears as a trough. It was obviously manifested that the content of Ni in the reverted austenite is higher than the matrix-tempered martensite. Considering the chemical composition of nickel contents, we may conclude that the reverted austenite is formed by a diffusional process of the alloying elements, particularly nickel, during the tempering process.

**Figure 8:**
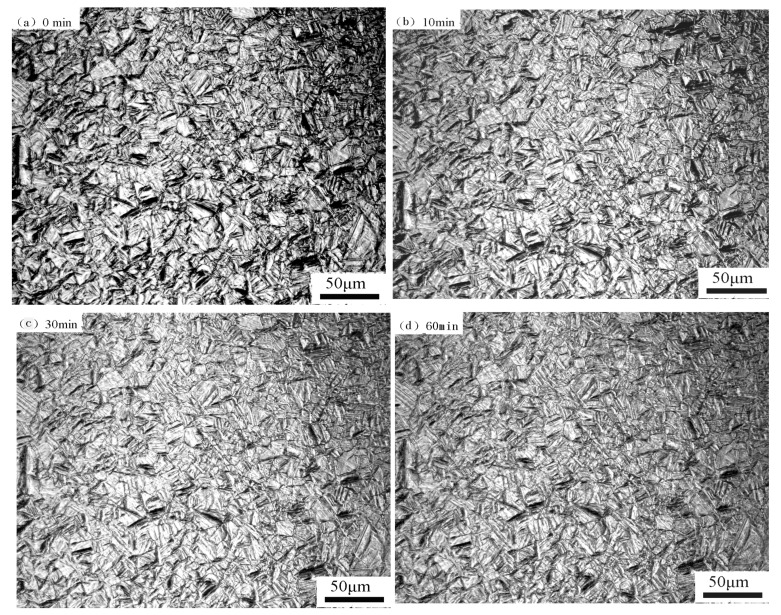
In situ observation of reverted austenite transformation of the 13Cr supermartensitic stainless steel tempered at 620 °C for different times: (**a**) 0 min, (**b**) 10 min, (**c**) 30 min, (**d**) 60 min.

Figure 8 represents the microstructure evolution after the quenching and tempering of 13Cr supermartensitic stainless steel at 620 °C for different times. Figure 8 shows a series of images of the reverted austenite transformation in situ observation of the 13Cr supermartensitic stainless steel tempering at 620 °C for 0~60 min. There is no obvious change in the microstructure from martensite to reverted austenite and the volume change was not observed to be associated with the transformation of acicular austenite [37]. It is indirectly manifested that the martensite conspicuously transformed to reverted austenite is a diffusion mechanism. Firstly, the volume shrinkage during martensite transformation to reverted austenite is slow in the tempering heating and holding processes. Secondly, the reason for this is that the surface relief is different from martensite transformation and there is no high-density dislocation. Therefore, it is considered that the formation of the reverted austenite should be generated by the nucleation growth method related to diffusion [30], not the diffusionless shear mechanism. The result of the reverted austenite transformation mechanism in our study is consistent with Lee’s research [22]. It is possible that Ni and C austenite stabilization elements gradually diffused to the reverted austenite at the temperatures for a long time. In the previous research work [37], carbon elements were preferentially positioned in the reverted austenite and alloy carbides failed to precipitate in the martensite during tempering, which meant that the martensite was unable to decompose. Subsequently, coalescence of the reverted austenite appeared.

Such a gradual reversion behavior is characteristic of a diffusional process [42]. The stability of reverted austenite depends on the Ni contents concentration of the reversed austenite. At a shorter holding time, Ni atoms are enriched in the reverted austenite, which increases the stability of reverted austenite and increases the volume fraction of reverted austenite. Therefore, the diffusion and redistribution of Ni between reverted austenite and the matrix are responsible for the reverted austenite transformation and growth [37]. The reverted austenite that showed a plate-like shape acts as the core of nucleation and reverted austenite formation is accompanied by the Ni diffusion process. It is revealed that the growth kinetics of the reverted austenite are dominated by the diffusion of Ni in the austenite and martensite phases, particularly in the austenite phase due to its relatively higher diffusion contents [35]. Martensitic shear reversion is characterized by a rapid change that only depends on the temperature change, but not on the time. It was known that the transformation thermodynamics are the temperature gradient, as is the fact that austenite formation is diffusion driven. The tempering time was focused on the influence it had on the reverted austenite above the As point tempering in the steel. Therefore, the mechanism through which the reverted austenite transformed from martensite is a diffusion mechanism.

## 4. Conclusions

In this paper, there are several conclusions obtained based on the results provided by X-ray diffraction and SEM with EBSD techniques in order to investigate the microstructure evolution of the reverted austenite and tempered martensite in the present work. The following conclusions can be drawn according to the research in this paper:

1. The microstructure mainly consists of tempered martensite and reverted austenite in the supermartensitic stainless steel. The amount of reverted austenite at room temperature increases and the micro-hardness correspondingly decreases during tempering at 620 °C for 1 h to 32 h. The results suggest that the contents of reverted austenite have an effect on the mechanical properties;
2. The reverted austenite nucleates uniformly and transforms at sub-block boundaries and prior grain austenite boundaries. As the tempering time increases, the low angle grain boundaries decrease and the dislocation density also exhibits a dramatic decline compared to the martensite matrix of tempering at 620 °C for 2 h to 32 h. The orientation relationship between reverted austenite and martensite is (111)γ//(011)α and [1$\bar{1}$0]γ//[1$\bar{1}$1]α, which meets the K–S relationship and the deviation angle is mainly concentrated at about 2 degrees;
3. The transformation mechanism of martensite to reverted austenite is formed by a diffusional process. Considering the chemical composition of nickel contents, it is concluded that the reverted austenite is formed by nickel diffusion during the tempering process. It revealed that the growth kinetics of the reverted austenite are dominated by the diffusion of Ni contents and there is no shear of the martensite matrix in situ observation by HTLSCM.

## Figures and Tables

**Table 1:** Chemical composition of the supermartensitic stainless steel (wt%).

C	Mn	Si	P	S	Ni	Cr	Cu	Mo	V	Ti	Nb
0.03	0.32	0.28	0.011	0.001	5.8	12.5	0.085	2.05	0.054	0.004	0.003
